# Young women’s joint relationship, sex, and contraceptive trajectories: Evidence from the United States

**DOI:** 10.4054/demres.2020.42.34

**Published:** 2020-06-03

**Authors:** Bridget Brew, Abigail Weitzman, Kelly Musick, Yasamin Kusunoki

**Affiliations:** 1 University of Mary Washington, Fredericksburg, VA, USA.; 2 University of Texas at Austin, USA.; 3 Cornell University, Ithaca, New York, USA.; 4 University of Michigan, Ann Arbor, Michigan, USA.

## Abstract

**OBJECTIVE:**

We identify common patterns of joint relationship, sex, and contraceptive trajectories in young adulthood and assess how selection into these trajectories differs across socioeconomic and demographic groups and varies with earlier sexual and reproductive experiences and attitudes.

**METHODS:**

We draw on a weekly panel of 581 young adult women in the United States that includes granular data on sexual and contraceptive behaviors. We use sequence analysis to describe joint relationship, sex, and contraceptive trajectories over the course of a year and multinomial logistic regression to examine how these trajectories are associated with socioeconomic disadvantage and minority racial status.

**RESULTS:**

We identify six trajectories characterized by differences in relationship stability, sexual regularity, and contraceptive efficacy. Many women report no romantic relationships over the year. Among those who do, instability in relationships, sex, and contraception is common. Less advantaged women are more likely to be on trajectories marked by frequent relationship transitions, coresidence, and less effective contraception. These socioeconomic differences are largely explained by earlier experiences and attitudes. Black women are the most likely to be on a trajectory characterized by simultaneous relationship, sex, and contraceptive instability, and this holds net of earlier experiences and attitudes.

**CONTRIBUTION:**

We provide a novel way of understanding how women’s relationship, sexual, and contraceptive trajectories co-evolve and vary by sociodemographic characteristics. Results highlight that instability is common in the young adult years but that differences in how trajectories unfold suggest greater risk of unintended pregnancies for socially disadvantaged and black women.

## 1. Introduction

Young adulthood is a pivotal period of the life course in which pathways to family formation and future economic advantage and disadvantage begin to solidify (Arnett 2000; Graber, Brooks-Gunn, and Petersen 1996; Rindfuss 1991; Rindfuss, Morgan, and Swicegood 1988). Fertility rates at this life stage are high in the United States relative to Europe (Lesthaeghe and Neidert 2006), particularly among disadvantaged women, many of whom report their pregnancies as unintended (Musick et al. 2009; Finer 2010). Prior research has documented sociodemographic differences in key proximate determinants of fertility, including the seriousness of relationships, sexual frequency, and contraceptive use (Kusunoki et al. 2016; Kusunoki and Upchurch 2011; Sassler, Michelmore, and Holland 2016). Building on this literature, we focus on the joint progression of these pregnancy-related behaviors over the transition to adulthood, identifying relationship, sex, and contraceptive trajectories and the characteristics of young women that map onto them. Our approach sheds new light on patterns that characterize the transition to adulthood and give rise to early and unintended pregnancy.

Relationship status, sexual frequency, and contraceptive use represent overlapping yet distinct dimensions of young women’s pregnancy risk. Sexual intercourse frequently occurs before marriage and outside of cohabiting or married relationships (Bearak 2014; Lichter, Anderson, and Hayward 1995; Musick 2007; Sassler 2010), with varying degrees of contraceptive efficacy and consistency (Avery and Lazdane 2008; England et al. 2016; Higgins, Hirsch, and Trussell 2008), especially among young adults (Kusunoki and Upchurch 2011; Levinson 1986). Given high levels of early and unintended childbearing in the United States and associations with subsequent disadvantage (e.g., McLanahan 2004), a robust body of research has developed to assess pregnancy risk. Prior literature, however, has tended to assess critical dimensions of pregnancy risk separately: for instance, examining how cohabitation, sexual initiation, and contraceptive use vary with attitudes, intentions, demographic background, family characteristics, and neighborhood context, but not with each other (Browning, Leventhal, and Brooks-Gunn 2005; Heinrich 1993; Littlejohn 2012; Sassler 2004). This has begun to change with studies emerging over the last decade that focus on associations between contraceptive use and sexual desire, sexual activity, and relationship characteristics such as seriousness and violence (Barber et al. 2013; Bergmann and Stockman 2015; Kusunoki and Upchurch 2011; Kusunoki et al. 2018). These newer studies take seriously the intertwined nature of relationship dynamics, sex, and contraceptive use, providing valuable insights into the ways in which they are linked. Nonetheless, the standard approach of treating one dimension as an outcome of another in a discrete-choice or event-history model provides an incomplete picture of the patterning of these dimensions over time.

We take a novel approach in this study, using sequence analysis to describe the joint progression of relationships, sex, and contraceptive use over a critical period of the life course. Instead of isolating a single event or attempting to demonstrate cause and effect, sequence analysis highlights how processes evolve together (Pollock 2007), making it a powerful tool for understanding the complexities that characterize early family formation and family life (Aassve, Billari, and Piccarreta 2007; Aisenbrey and Fasang 2017; Billari 2001; Frye and Trinitapoli 2015; Killewald and Zhuo 2019). We draw on the Relationship Dynamics and Social Life Study (RDSL), an innovative weekly panel of 581 young adult women in one county in the United States that includes rich, granular data on women’s sexual and contraceptive behaviors and other background information. To our knowledge, no study has applied sequence analysis to illuminate joint trajectories of young adults’ relationships, sexual activity, and contraceptive use, leaving open important questions about how these key demographic behaviors cluster together in ways that set the stage for future relationships, family life, and related economic constraints and opportunities (Meier and Allen 2009). Although data come from one county in the United States, the characteristics of young women in the RDSL reflect the US population of this age on key characteristics (Ela and Budnick 2017; Clark 2018). Moreover, what we uniquely learn from these intensive panel data informs a broader understanding of how young adult relationships evolve over the course of this critical life transition.

## 2. The joint progression of young adult relationships, sexual activity, and contraceptive use

Relationships, sex, and contraceptive use are inextricably linked. More fully accounting for the interconnected nature of decisions along these dimensions is important to better understanding the complex ways in which they relate to family formation. For instance, relationship dynamics contribute to sexual desire and frequency (Higgins et al. 2008; Higgins, Tanner, and Janssen 2009; Tavory and Swidler 2009). Young women may have sex more regularly with their partners when they are in more serious relationships, particularly if this seriousness contributes to stronger desires for pregnancy (or at least less opposition to it) (Weitzman et al. 2017). Women also have sex more regularly with their partners when they live together (Rhoades, Stanley, and Markman 2012). Even when women do not want to have sex, some may consent to unwanted sex in an effort to please their partners, avoid conflict, or maintain a sense of intimacy in their relationships (Aarons and Jenkins 2002; O’Sullivan and Allgeier 1998). This also implies that in some cases, not having sex may result in undesirable relationship dynamics or even relationship termination. Some women may further be coerced into unwanted sex when in abusive relationships (Campbell 2002).

Sexual relationships are often accompanied by implicit or explicit decisions about contraceptive use ‒ decisions that may be challenging to navigate. For example, couples can be conflicted about whether or not they desire pregnancy and/or eroticize the risk of pregnancy even when they do not want one, and this ambivalence can lead to inconsistent contraceptive use (Higgins et al. 2008a; Miller, Barber, and Gatny 2013). Moreover, even when two people intend to prevent pregnancy, they may have misgivings about coital contraception because they view it as amoral, a hassle, a barrier to pleasure and intimacy, or a sign of mistrust (Higgins et al. 2009; Tavory and Swidler 2009). Individuals may have further concerns about hormonal contraception if they view it as unnatural, requiring too much effort, a historical tool of racialized population control, or repressing their sexual desire (Barber, Yarger, and Gatny 2015; Higgins et al. 2008b). And yet, if individuals do not use hormonal or barrier contraception, they must choose either an alternative, less effective method like withdrawal, abstain from vaginal intercourse, or accept the risk of potential outcomes including pregnancy. Contraceptive decisions can therefore not only stem from but also affect decisions related to sex.

Relationship dynamics further shape how concerns and misgivings about contraception play out (Kusunoki and Upchurch 2011; Manlove et al. 2011). For example, when contraception is viewed as an impediment to pleasure and intimacy, it may also be perceived as a detriment to relationship stability and well-being (Aarons and Jenkins 2002; Brady et al. 2009; Tavory and Swidler 2009). Women who do not want to become pregnant and do not want to use contraception but who view sex as a critical component of relationship maintenance must therefore make difficult choices about whether to abstain from vaginal intercourse, and if not, whether to use contraception. These difficulties may result in inconsistent behaviors over time.

## 3. Correlates of young women’s relationship, sexual, and contraceptive behavior

Relationship formation, sexual activity, and contraceptive use are strongly patterned by sociodemographic characteristics in the United States. Women with lower socioeconomic status have more serious relationships in young adulthood (Kusunoki et al. 2016) and transition to cohabitation faster but also experience less stable coresidential relationships, on average, than do their more advantaged counterparts (Sassler, Michelmore, and Holland 2016). In addition, they tend to experience more unintended pregnancies and become parents earlier in the life course (Bumpass and Lu 2000; Carlson and Furstenberg 2006; Musick et al. 2009; Rindfuss and St. John 1983; Sassler, Michelmore, and Holland 2016; Upchurch, Lillard, and Panis 2002). These differences suggest that the overall pattern of young women’s joint relationship, sex, and contraceptive trajectories will vary by socioeconomic status, with lower status women spending more time in sexual, cohabitating relationships with less effective contraception and/or transitioning in and out of these relationships more frequently than women with comparatively higher status.

US research on the link between socioeconomic backgrounds and sexual relationships tends to focus on either disadvantaged groups or college students. In their work on low-income women, Edin and colleagues (Edin and Kefalas 2005; Edin et al. 2007) document ambivalence toward pregnancy and inconsistency in contraceptive use among young mothers. They argue that low avoidance attitudes toward early motherhood among these women result from poor education and career prospects that provide few alternate sources of meaning. Meanwhile research on college students documents a hook-up culture in which sexual relationships outside of long-term, stable partnerships are common but decoupled from childbearing (England, Shafer, and Fogarty 2008; Paul and Hayes 2002). Because these streams of research focus on women at opposite ends of the socioeconomic distribution, it remains unclear how relationship dynamics compare by socioeconomic status. For example, should we expect a similar level of instability in young adult relationships by socioeconomic status? Do key differences between the more and less advantaged rest primarily on contraceptive use, or are there differences as well in the patterning and nature of their relationships at this life stage? We address these questions by following the joint trajectories of relationships, sexual activity, and contraceptive use in a socioeconomically diverse sample of young women.

In the United States, relationship and reproductive behaviors are likely to vary by race as well as socioeconomic status, in part because black populations tend to be poorer than white populations. Indeed, black women are more likely than white women to experience an unintended pregnancy (Finer and Zolna 2016), partially because they use contraception less frequently and rely on less effective contraception than white women (Dehlendorf et al. 2014). Black women are also more likely to have a baby outside of a married or cohabiting union (Raley 2001) and are less likely than their white counterparts to ever marry (Lichter et al. 1992). In the county in which our study is based, black women express more negative views of contraception (Barber, Yarger, and Gatny 2015) and spend less time in relationships and have sex less frequently (Kusunoki et al. 2016) than do white women but otherwise report comparable numbers of partners and rates of contracepting consistently (Kusunoki et al. 2016). Evidence thus suggests that black women’s transitions to adulthood will be characterized by shorter duration sexual relationships and more time outside of relationships. Following the joint progression of relationships, sex, and contraception, as we do in this study, will shed light on distinct patterns of relationships, sex, and contraception common among black women, including time spent out of relationships.

The life course perspective (Elder 1995) indicates that beyond individuals’ demographic background, early sexual and reproductive experiences influence subsequent relationship, sexual, and contraceptive behavior during the transition to adulthood. For example, sexual initiation before the age of 15 is associated with more sexual partners and an increased likelihood of inconsistent contraceptive use in adolescence and beyond (Santelli et al. 1998; Magnusson, Masho, and Lapane 2012). Early sex and more sexual partners during adolescence should be associated with more time in sexual relationships and greater instability in sexual relationships during early adulthood, as well as less effective contraceptive use. Further, we know that early, unintended pregnancies are associated with subsequent unintended pregnancies (Guzzo and Hayford 2011), suggesting inconsistent contraception in either ongoing or new relationships. Early pregnancies resulting in birth may also reduce women’s time and commitment to romantic relationships or, on the contrary, enhance relationship stability for some.

One explanation for why earlier life experiences shape subsequent behavior is that these experiences affect the formation of attitudes, expectations, and intentions. For instance, women who have sex without contraception in adolescence are more likely to expect to have sex without contraception as young adults (Barber, Yarger, and Gatny 2015). Moreover, studies from the United States and Europe find that expectations and intentions are strong predictors of subsequent fertility-related behaviors (Miller, Barber, and Gatny 2013; Rosengard et al. 2004; Thomson 1997; Williams, Abma, and Piccinino 1999). For example, women with a preference against having a child outside of a marriage take precautions to prevent that experience, including delaying intercourse until marriage and consistently using contraception while unmarried (Shattuck 2019). This leads us to expect that young women with more positive attitudes toward nonmarital sex will spend more time in sexual relationships and potentially transition in and out of sexual relationships more frequently. Likewise, women with more positive attitudes toward contraception will be more likely to use contraception effectively in sexual relationships, and conversely young women who view early motherhood favorably and who want an early pregnancy will spend a greater share of their transition to adulthood in sexual, cohabiting relationships with ineffective contraception.

## 4. Data and methods

### 4.1 Relationship dynamics and social life study

The RDSL is a random, population-representative panel of young women who were age 18 or 19 and residing in one Michigan county at the time of study recruitment. Participants were randomly selected from the Michigan driver’s license and state ID card registry with a response rate of 84% (94% of those who were successfully located) (Barber et al. 2016). This sampling frame is 96% aligned with census projections for 18- and 19-year-old women in the county (Barber, Kusunoki, and Gatny 2011). Though geographically restricted, the RDSL sample mirrors nationally representative samples of women of comparable age with respect to birth and marriage rates, residential arrangements, educational enrollment, and employment rates (Clark 2018; Ela and Budnick 2017). However, when compared with nationally representative samples like the American Communities Survey, African American women are approximately twice as represented and Hispanic women half as represented in the RDSL (Clark 2018).

A unique strength of the RDSL data is that they provide detailed, prospective information on the interrelated dimensions of pregnancy risk in early adulthood. Respondents first completed a 50-minute in-person, baseline interview that included demographic background and sexual and reproductive history and attitudes. At the close of baseline, respondents were invited to participate in five-minute weekly surveys, or ‘journals,’ that collected information about their relationships and sexual and contraceptive behaviors online or through an automated phone program for the next two and a half years (Barber et al. 2016).^5^ For the journal portion of the study, 99% of respondents agreed to participate, completing these weekly journals an average of eight days apart (Barber et al. 2016). Our sequence analysis relies on the information collected through these journals. Given their temporal regularity, we refer to our unit of analysis as person-weeks. Having multiple weeks nested within each respondent uniquely enables us to follow women as they transition in and out of relationships, even very short relationships.

The full RDSL journal sample includes 992 women. To ensure that all respondents in our analytic sample remained in the study long enough and participated consistently enough for us to observe changes in relationships and contraception, we limit our analytic sample to those who remained in the study for at least one year, completed at least 12 journals (thus averaging no less than 1 journal per month), and had no more than a three-month gap between journals (*N* = 631 women). Because our primary interest is the joint progression of relationships, sex, and contraceptive use, all of which may be affected by pregnancy, we further restrict the sample to women who were not pregnant at baseline and did not become pregnant before completing 12 journals (*N* = 581). If a respondent became pregnant after 12 journals, she remained in our sample, but her sequence was censored starting at the time she first reported being pregnant.

Our final analytic sample thus includes journals collected in the first year of the study, for a total of 22,389 person-weeks from 581 respondents. Appendix Table A compares patterns of response across our analytic sample and the overall sample of RDSL journals and respondents. Appendix Table B compares the descriptive statistics of the two samples. Notably, minority and lower socioeconomic status respondents tended to leave the RDSL study earlier and completed fewer journals on average than did other respondents (Barber et al. 2016). Robustness checks, discussed in detail below, indicate that the main results of our sequence analysis are not sensitive to changes in our sample restrictions, including our treatment of pregnancy, reporting gaps, sequence lengths, and journals beyond the first year of the study.

### 4.2 Measures

#### Relationships, sex, and contraceptive use.

We begin by constructing measures that jointly capture respondents’ relationship, sexual, and contraceptive states as reported in each journal entry. We define three relationship states: not in any kind of relationship, in a non-coresidential relationship, and in a coresidential relationship. These states are based on information from a set of weekly questions about women’s relationship status,^6^ including whether the respondent was married, engaged, in a “special romantic relationship,” or in “any type of relationship that involves physical or emotional contact.” When a respondent reported being in any kind of relationship, she was asked whether she had a “place [to] live that is separate from where [her partner] lives.” We define respondents as coresiding in weeks when they reported being in any kind of relationship and not having a separate residence from their partner.^7^

In weeks when respondents reported being in any kind of relationship, they were also asked whether they had vaginal intercourse with that partner. Responses to this question allow us to define two states of sexual activity. That is, respondents are defined as sexually active in weeks when they reported being in a relationship and having intercourse and as not sexually active when they were either not in a relationship or in a relationship with no reported intercourse that week.

In all weeks (irrespective of relationship status) respondents were asked “Did you use or do anything that can help people avoid becoming pregnant, even if you did not use it to keep from getting pregnant yourself?” When a respondent answered “yes,” she was then asked a series of follow-up questions about noncoital specific methods, including oral contraceptive pills, the patch, NuvaRing, Depo-Provera, implant, IUD, and rhythm method. In weeks when respondents reported sexual intercourse, they were also asked whether they used “some method of birth control every time [they] had intercourse.” If so, they were then asked a set of questions about coital-specific methods including condoms (male and female), diaphragm/cervical cap, spermicide, and withdrawal. Because contraceptive methods vary in effectiveness (Sundaram et al. 2017), we differentiate between two contraceptive states based on responses to these questions: less effective/inconsistent (unprotected sex; methods with high failure rates, including diaphragm, spermicide, withdrawal, and rhythm; and inconsistent condom use) and effective/consistent (long-acting and hormonal methods and/or consistent condom use). While some studies distinguish less effective methods from no method (e.g., Glei 1999), we combine no contraception and less effective contraception because both practices put women at risk of becoming pregnant (Hatcher et al. 2007; Sundaram et al. 2017). A robustness check with different states for weeks when respondents used no method and less effective methods is consistent with our main results; this and other robustness checks are discussed in detail below.

We cross-classify relationship, sex, and contraceptive states to generate the following joint states: (1) not in a relationship (by definition did not have sex, as sexual partners are considered a type of relationship); (2) in a non-coresidential relationship, did not have sex; (3) in a non-coresidential relationship, had sex using less effective contraception; (4) in a non-coresidential relationship, had sex using effective contraception; (5) in a coresidential relationship, had sex using less effective contraception; and (6) in a coresidential relationship, used effective methods to avoid pregnancy (including no sex because of the relative rarity of living with a partner and reporting no sex in a given week).

#### Demographic background.

We examine four indicators of respondents’ sociodemographic background. The first is a composite measure of childhood disadvantage, based on whether the respondent’s mother had her first child in her teens; her mother did not complete high school; she grew up in a family structure other than two-parent; and her family received public assistance during her childhood. We consider respondents without any of these circumstances to have low disadvantage, respondents with one of these circumstances to have medium disadvantage, and respondents with two or more of these circumstances to have high disadvantage. Our second sociodemographic measure is race, measured with a dichotomous indicator of whether a respondent identifies as black or not.^8^

We also include respondents’ employment status and school enrollment at baseline. Respondents are considered employed if they work any hours for pay. School enrollment is captured with three categories: incomplete high school (dropped out or still enrolled); graduated high school and not enrolled in post-secondary education; and enrolled in any type of post-secondary education, including vocational school, community college, and four-year college.

#### Sexual and reproductive history.

Measures of sexual and reproductive history include whether a respondent first had vaginal intercourse at age 14 or younger; number of sexual partners by baseline (0, 1, 2 to 3, 4 or more); and whether she had a pregnancy prior to baseline (recall that if a woman was pregnant *at* baseline, she is excluded from the sample).

#### Attitudes toward sex, contraception, and fertility.

We examine three sets of attitudinal measures that could contribute to women’s joint relationship, sex, and contraceptive trajectories. The first is her attitude toward premarital sex, which was assessed by asking how much she agreed or disagreed with the statement “Young people should not have sex before marriage.” Possible answers ranged from (1) strongly agree to (5) strongly disagree. We collapse responses into a dichotomous indicator, where (1) equates agree or strongly agree and (0) otherwise.

The second measure addresses attitudes about contraception and is derived from respondents’ baseline agreement on a 5-point scale with the following statements: “If a woman asks her partner to use a condom, he will think that she doesn’t trust him”; “Birth control is morally wrong”; “In general, birth control is too much of a hassle to use”; “Using birth control is likely to make a woman feel sick”; “Using birth control interferes with sexual enjoyment”; “If a girl uses birth control, she is looking for sex”; “In general, birth control is too expensive to buy”; and “It takes too much planning ahead of time to have birth control on hand when you’re going to have sex.” We sum responses to form a contraceptive opposition scale, which ranges from 0 to 40, with higher values indicating more opposition. Based on the summary statistics of this measure, we create four categories, approximately reflecting quartiles of opposition: low (0 to 5), mild (>5 and ≤ 8), moderate (>8 and ≤ 10), and high (>10).

Finally, the third set taps respondents’ attitude toward fertility, which we capture with two measures. One is a Likert scale of whether a respondent believes that “relationships between men and women improve after they have a baby together,” with responses ranging from 1 to 4 where higher numbers indicate stronger agreement. We dichotomize this measure; those that agree or strongly agree are coded as a 1. The other is respondents’ desire for pregnancy, that is, how much they reported “wanting to get pregnant” at baseline. Possible values range from 0 to 5 with 5 being the strongest desire. The vast majority of respondents had no desire. Thus, we dichotomize this measure such that respondents with any desire (>0) are coded as desiring pregnancy.

#### Additional control.

We include one additional control variable (not shown in tables, but available on request). Because religious beliefs may shape moral perceptions of sex and contraception, we include a dichotomous measure of religiosity. Respondents who say that their religious faith is “very important” or “more important than anything” are coded as religious (57% of the analytic sample); respondents who say their religious faith is somewhat or not important are coded as not religious.

### 4.3 Methods

#### Sequence analysis.

Sequence analysis is an umbrella category of techniques that are used to address questions related to the temporal ordering of events (Abbott 1995). The first stage of our analysis is to create sequences for each respondent by stringing together their joint relationship-sex-contraception statuses as reported in their (approximately) weekly journals over the course of one year. Because every respondent can have a unique sequence, the next stage of our analysis separates sequences into meaningful groups in order to compare trajectories across women (Aisenbrey and Fasang 2010; Brzinsky-Fay and Kohler 2010). We use optimal matching (OM) techniques that calculate the cost of turning one sequence into another sequence based on element substitutions, insertions, and deletions (Abbott 1995; Brzinsky-Fay and Kohler 2010; Lesnard 2010).

For our OM substitution costs we use the mean of the transition probabilities between neighboring elements and rely on the default indel (insertions and deletions) cost of one. Because the lengths of respondents’ sequences vary based on the number of journals they completed in the year of observation,^9^ we standardize comparison pairs based on sequence length to limit the impact of disparate sequence lengths on transformation costs (Brzinsky-Fay, Kohler, and Luniak 2006). The final step of our sequence analysis is to use the dissimilarity matrix that results from OM in order to cluster like sequences. Generally, cluster analysis uses similarity measures in the data to unearth patterns (Jain, Murty, and Flynn 1999). For this stage we use Ward’s linkage, which is a hierarchical method that minimizes variance within clusters (Blashfield 1976). We further rely on the Duda and Hart stopping rules to identify the number of clusters that are the most distinct from each other (Everitt and Rabe-Hesketh 2006). All steps of the sequence analysis are performed with the Stata 14.0 SQ package.

#### Multivariable analyses.

After identifying common sequences of joint relationship-sex-contraception states among respondents, we assess heterogeneity in clusters by sociodemographic background, earlier adolescent sexual and reproductive experiences, and attitudes toward sex, contraception, and fertility. Because sociodemographic background may shape both the clusters that women sort into and their early sexual and reproductive experiences and attitudes, we estimate two multinomial logistic regression models in succession. In the first (Model 1), we include measures of childhood disadvantage and race. In the second (Model 2), we add measures of sexual and reproductive history and attitudes toward sex, contraception, and fertility. All models control for religiosity.

## 5. Results

### 5.1 Descriptive characteristics of our sample

Table 1 shows our joint relationship, sex, and contraceptive states pooled over person-weeks. These joint statuses form the building blocks of our sequence analysis. The modal category is not in a relationship (39% of person-weeks). The next most common category is in a non-coresidential relationship with no sex (26% of person-weeks). Thus about two-thirds of person-weeks involved no sexual relationship. Another 15% are in non-coresidential relationships that included sex using effective contraception. The remaining 20% of person-weeks are distributed across the remaining three joint relationship, sex, and contraceptive states involving either coresidential relationships or less effective contraception.

Descriptive statistics on sociodemographic variables, prior experiences, and attitudes are provided in Table 2. Our sample is 32% disadvantaged by our measure and 27% black. Half of respondents are employed, and two-thirds are enrolled in post-secondary education. An estimated 12% had first sex at age 14 or younger, and two-thirds reported at least one sexual relationship by baseline. There is substantial variation in attitudes about sex, contraception, and fertility, with the exception of very little pregnancy desire.

### 5.2 Description of sequences and clusters

**Figure 1:**
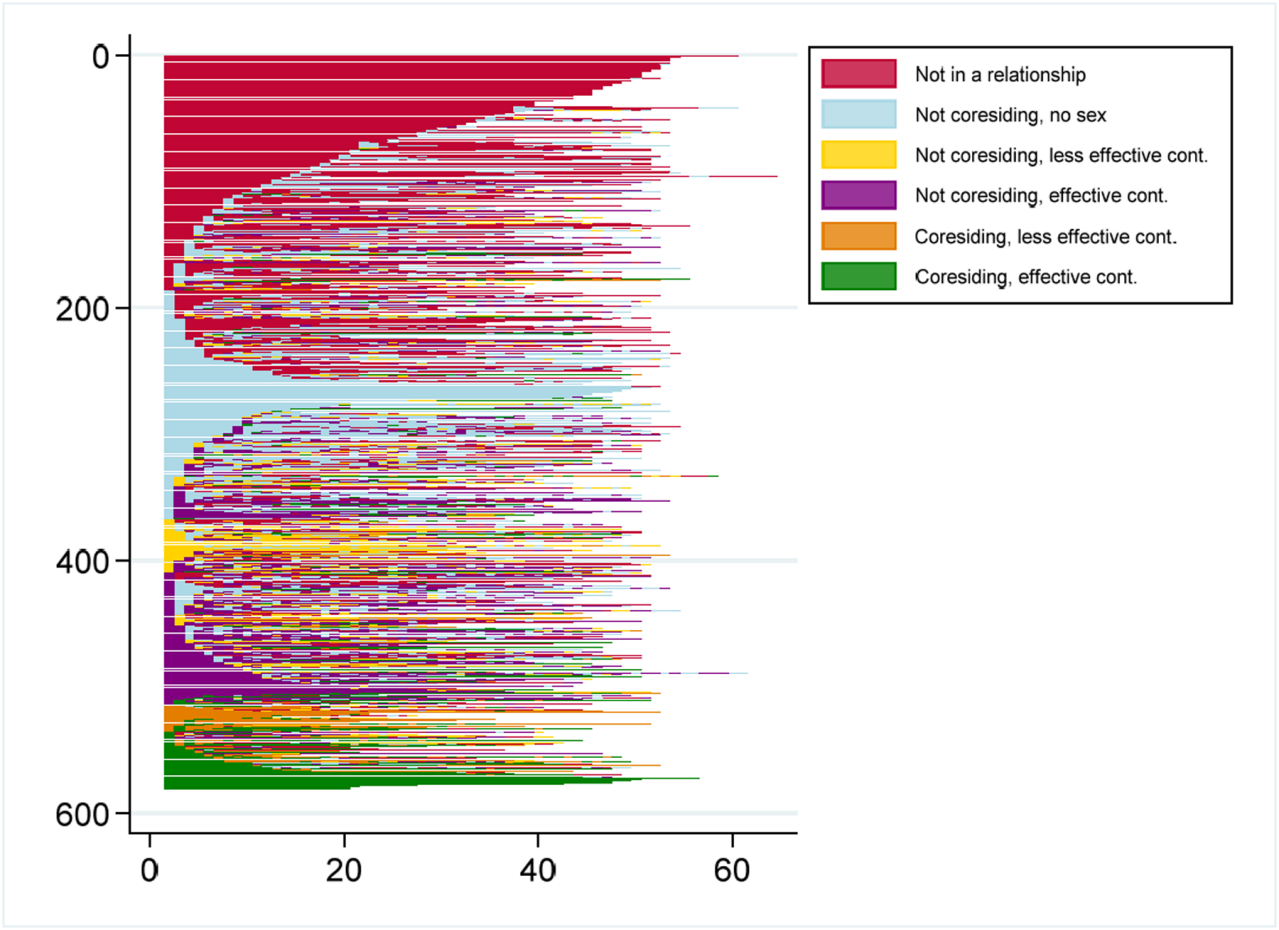
Sequences of joint relationship, sex, and contraceptive states in the first year of the RDSL, N = 581 *Note:* Each row depicts the sequence of an individual woman.

Figure 1 shows respondents’ sequences; each horizontal line is a sequence of the respondent’s weekly relationship-sex-contraception states strung together in temporal order. The relationship-sex-contraception states are called elements. A multiweek period without changing states is an episode. We refer to every change in state – whether following a single week or a multiweek episode – as a transition. Higher numbers of elements and episodes mean more transitions between states and are thus both indicators of sequence complexity (Elzinga 2010). We refer to frequent changes in status as instability regardless of whether they are changes in sexual activity, contraceptive use, or relationship status. It is important to note that we do not identify partner changes. Identifying particular partners adds a level of detail that goes beyond the scope of this analysis. Without accounting for partner change from week to week, we may overestimate instability in some instances, such as when a woman is in a stable relationship and has sex with her partner one week, followed by a week when she does not have sex within the same partnership. In select instances we may also underestimate instability, as when a woman changes non-coresidential partners between weeks while maintaining the same sexual and contraceptive practices.

At the very top of Figure 1, in pink, are respondents who report no relationships in the first year of the RDSL. Just below are those who began the study with no relationship and then transitioned in and out of other joint statuses, such as non-coresidential relationships without sex (in light blue) and coresidential relationships with effective contraception (in green). Toward the middle of the plot are sequences that begin in light blue, yellow, and purple, representing women who began the RDSL in non-coresidential relationships (without sex, with less effective contraception, and with effective contraception, respectively). Finally, toward the bottom are the few sequences beginning in orange and green, representing respondents who began the RDSL in coresidential relationships with less effective and effective contraception, respectively. As can be seen in this figure, approximately half of respondents either did not have sex or had sex only infrequently over the course of the year. Of the remaining respondents, most spent the majority of their person-weeks in and out of non-coresiding relationships, with varying degrees of sexual activity and contraceptive effectiveness. Only a few respondents spent the majority of the first year of the RDSL study coresiding.

**Figure 2:**
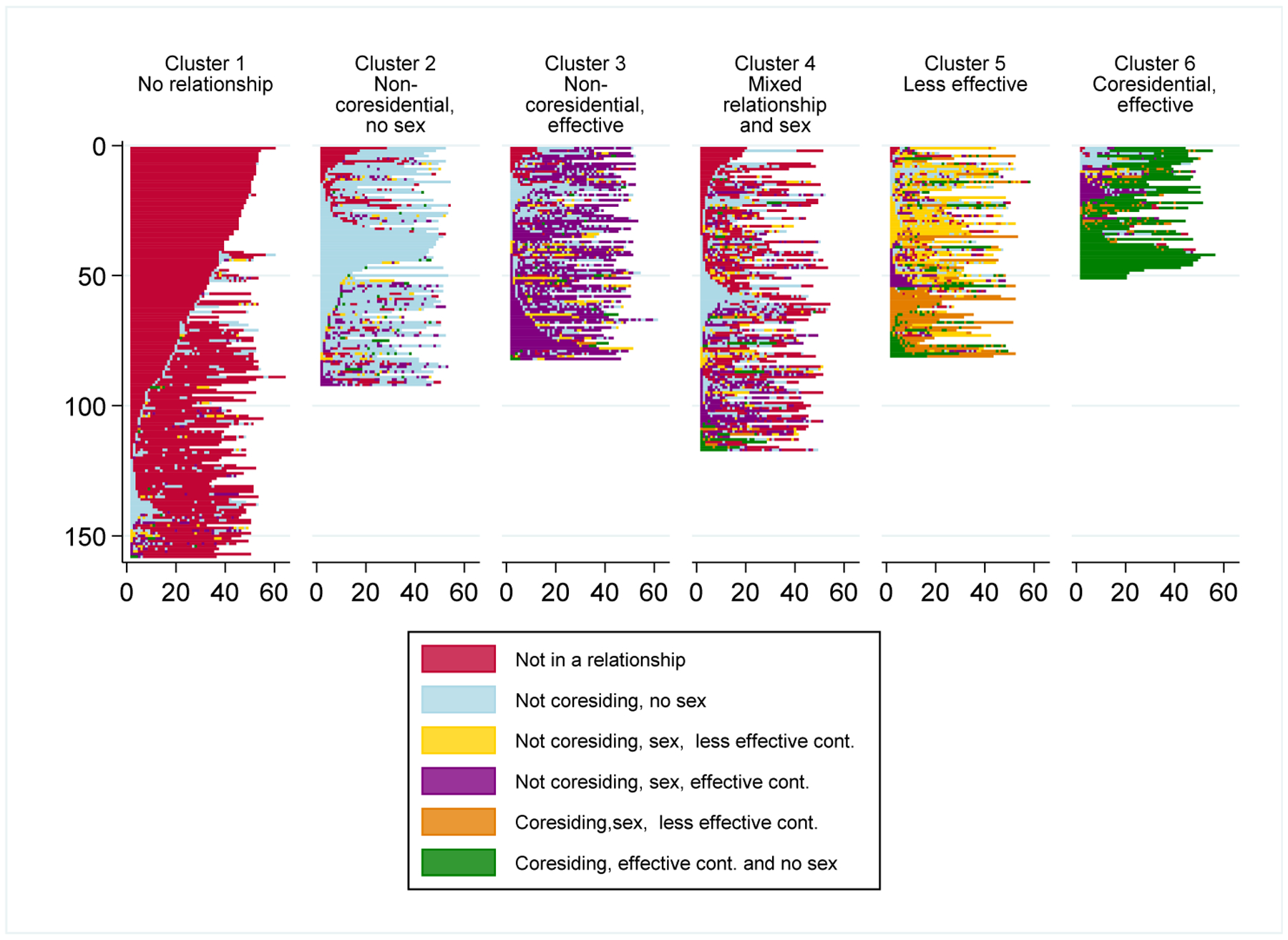
Six clusters of relationship-sex-contraception sequences in the first year of the RDSL, N = 581 *Note:* Each row within each cluster depicts the sequence of an individual woman. Clusters are mutually exclusive and exhaustive, that is, each woman is included in one cluster only.

Our cluster analysis more formally compares sequences across women by grouping like sequences of young women’s joint relationship, sex, and contraceptive states together. We identify six clusters (based on a Duda and Hart index of 0.96 and a pseudo T-squared of 5.27) that represent similarities in the order and complexity of these joint states, as measured by the number of distinct elements and number of episodes. The ideal typical sequence for each cluster (Kohler, Luniak, and Brzinsky-Fay 2016) is included as Appendix Figure C1 and the fraction of person-weeks in each sequence state by cluster is shown in Appendix Figure C2. Figure 2 is the visual portrayal of like sequences grouped into our six-cluster solution; Table 3 further describes characteristics of the sequence clusters.

The first cluster (“no relationship”) characterizes 27% of women, making it the most common cluster. Respondents in this cluster were not in any type of relationship for fully 89% of their combined person-weeks (Table 3), as can be seen by the preponderance of pink elements in the first panel of Figure 2. Women in this cluster also experienced the fewest transitions between states across person-weeks, as evidenced by the lowest number of elements and episodes on average (2.07 and 4.82, respectively, Table 3).

The second cluster (“non-coresidential, no sex”) describes women who spent the majority of the year in relationships that involved no coresidence or sex (76% of person-weeks, Table 3). Visually, this is conveyed by large swatches of light blue in the second panel of Figure 2. Women in this cluster averaged about the same number of elements as the first cluster, but with somewhat more instability as indicated by more episodes (2.70 elements and 8.51 episodes).

Women in the third cluster (“non-coresidential, effective”) spent the majority of their person-weeks in non-coresidential relationships, having sex, and using effective contraception (59%), as indicated by the majority purple elements in the third panel of Figure 2. Despite having one majority element, women in this cluster had the highest average number of episodes (14.77, Table 3), with transitions largely into the non-coresidential relationship without sex state.

The fourth cluster (“mixed relationship and sex”) has no single majority state and is characterized by a great deal of instability. This can be seen by the lack of a dominant color in the fourth panel of Figure 2, frequent transitions between states, and relatively high number of episodes (13.41, Table 3). In just over half of all person-weeks in this cluster, women reported being in non-coresidential relationships (i.e., no sex, 28%), less effective contraception (6%), and effective contraception (17%). A substantial minority of person-weeks were not in any relationship (44%), and only 28% included sexual activity, of which only a small share were in coresiding relationships (5% of person-weeks collectively).

Like the fourth cluster, the fifth cluster also has no single majority state. Most person-weeks in this cluster (“less effective”) are in one of the less effective contraception states (i.e., in a non-coresiding, 31%) or a coresiding (28%) sexual relationship, shown by the yellow and orange elements in the fifth panel of Figure 2. Women in this cluster experienced the highest mean number of elements over the year (3.94, Table 3), as well as frequent transitions between states (13.31 episodes). The high incidence of less effective contraceptive use makes this the highest risk group for pregnancy.

Finally, the sixth cluster (“coresidential, effective”) describes women who spent the majority of the year in coresiding relationships using effective contraception (71% of person-weeks). This includes less than 9% of respondents overall and is the smallest cluster. (Not having sex in a coresidential relationship is counted as effective contraception and is an uncommon combination.) Women in this cluster experience relatively few transitions with an average of 2.92 elements and 7.01 episodes in their joint sequences (Table 3).

In sum, our sequence analysis yields six distinct clusters that simultaneously characterize young women’s relationship, sex, and contraceptive experiences during the transition to adulthood. These patterns indicate that young women tend to either predominantly (1) not be in a relationship; (2) be in a non-coresidential relationship without sex; (3) transition frequently in and out of having sex in non-coresidential relationships while using contraception effectively; (4) transition frequently in and out of non-coresidential relationships with varying degrees of sexual activity and contraceptive effectiveness; (5) frequently rely on less effective contraception in sexual relationships (both coresidential and non); or (6) maintain coresidential relationships that typically involve sex and effective contraception.

### 5.3 Multivariable results

We next test how young women sort into the clusters identified above using multinomial logistic regression. Model 1 includes only childhood disadvantage, race, and our control for religiosity. Model 2 adds baseline educational attainment and employment, sexual and reproductive history, and attitudes toward sex, contraception, and fertility to this model. Full model results are shown in Appendix Table D.

Because multinomial results are cumbersome to interpret when they are expressed as odds of cluster membership relative to a baseline group, we focus our discussion on predicted probabilities. Table 4 shows predicted probabilities of cluster membership, derived from estimates in Appendix Table D, varying characteristics of interest and holding all others at their observed values. Table 5 summarizes information on the statistical significance of these contrasts, again drawing from model estimates.

#### Demographic background.

Results comparing the observed share in a given cluster to predicted probabilities derived from Model 1 (Table 4) suggest that women with low levels of disadvantage in this county are overrepresented in Cluster 1 (no relationships). For example, the first column of Table 4 shows the predicted shares in Cluster 1 by low, medium, and high childhood disadvantage, holding race constant: 32%, 23%, and 25%, respectively, relative to the overall observed share of women in Cluster 1 (27.2%). Women with low disadvantage appear overrepresented as well in Clusters 2 and 3, both characterized by non-coresidential relationships and either no sex or sex with effective contraception. By contrast, women with high levels of disadvantage appear overrepresented in Clusters 5 and 6, characterized more by coresidential relationships as well as less effective contraception. Differences by social disadvantage are smaller in Cluster 4 (also characterized by no relationships, although with more complexity). Socioeconomic differentials in these clusters are not monotonic (i.e., medium disadvantage is distinct). Black women in this county are overrepresented in Cluster 4 and underrepresented in Clusters 5 and 6.

Table 5 shows which contrasts across clusters are statistically significant, and the +/− in the table indicates the direction of difference. For example, the first cell in Table 5 reads “+4,5,” indicating that the relative odds of being in Cluster 4 or 5 compared to Cluster 1 are higher for respondents with medium disadvantage than for respondents with low childhood disadvantage. This table highlights two key findings from Model 1. First, women in this county with higher (versus low) levels of childhood disadvantage have significantly higher odds of being in Cluster 5 than in Clusters 1, 2, 3, or 4. In other words, social disadvantage is associated with being in the cluster characterized by less effective contraception. Whereas only 8% of women with low disadvantage fall into Cluster 5, the prevalence is more than double for women with medium (17%) and high disadvantage (20%, Table 4, Model 1).

The second key finding is that, net of social disadvantage, black women (versus non-black) have statistically significantly lower odds of being in this cluster (Cluster 5) and Cluster 6 (characterized by coresidential relationships) than Cluster 4. For example, 10% of black women fall into Cluster 5, compared to 16% of non-black women (Table 4, Model 1). When compared to their likelihood of being in Cluster 4, this suggests that black women are 64% less likely to be in Cluster 5 relative to Cluster 4 (.28–.10/.28), whereas non-black women are 6% less likely to be in Cluster 5 relative to Cluster 4 (.17–.16/.17). In sum, black women’s trajectories are characterized by more time spent transitioning in and out of relationships with varying degrees of sexual activity than non-black women’s trajectories. Although time out of sexual relationships is clearly associated with lower pregnancy risk, instability in relationships (evident in Cluster 4) may make it harder to establish consistent contraception and thus be associated with higher pregnancy risk.

Model 2 includes controls for baseline educational attainment and employment, sexual and reproductive experiences, and attitudes toward sex, contraception, and fertility. Notably, when including these controls, no significant differences by childhood disadvantage remain (Table 5). This suggests that the association between childhood disadvantage and cluster membership works largely through its association with young women’s educational status, early sexual and reproductive experiences, and attitudes. Racial differences, however, retain their statistical significance, thus suggesting that education, experiences, and attitudes explain little of the racial differences we observe in cluster membership.

#### Employment and education.

As can be seen in Model 2, employment status is not significantly associated with cluster membership, but educational status is. That is, relative to not having completed high school by age 18 or 19, being enrolled in post-secondary school at baseline is associated with significantly lower odds of being in Cluster 4 relative to Clusters 2 and 3. Young women who are enrolled in post-secondary school thus have lower odds of being on a complex trajectory that includes many transitions in and out of relationships, sex, and contraceptive use than of being on more stable trajectories in which they predominantly spend their time in non-coresidential relationships that are either not sexually active or sexually active but accompanied by effective contraceptive use (Table 5).

#### Sexual and reproductive history.

The results of Model 2 also show that a young woman’s sexual and reproductive history is predictive of her later joint relationship, sex, and contraceptive trajectory. However, early sexual debut and prior pregnancies are not among these significant predictors (Table 5, Model 2). Instead, the most consistent of the experience variables in predicting cluster membership is prior number of sex partners. Most notably, women who ever had sex before baseline (e.g., having one or more past partners versus never having had sex) have significantly lower odds of being in Clusters 1 and 2 than in Clusters 3, 4, 5, and 6 (Table 5, Model 2). That is, women with previous partners are less likely to be in clusters characterized by no sexual relationships (and conversely, those with no prior partners are less likely to be in clusters that include sex). For instance, among respondents with a history of at least four sex partners by baseline, an estimated 9% are in Cluster 1 and 10% in Cluster 2, relative to 56% and 21%, respectively, of those with no previous partners (Table 5, Model 2).

#### Attitudes toward sex, contraception, and fertility.

Women’s attitudes toward contraception and desire for pregnancy, but not their attitude toward premarital sex or belief that babies improve relationships, are predictive of cluster membership. A few key findings emerge: Opposition to contraception is positively associated with less effective contraceptive use. While only 9% of women with low opposition to contraception fall into Cluster 5, the prevalence is more than double for women with moderate (20%) and high opposition (19%, Table 4, Model 2). Women with higher opposition to contraception (moderate/high versus low) have higher odds of membership in Cluster 5 than in any of the other clusters (statistical significance depends somewhat on the particular contrast, but consistency is overall strong; Table 5, Model 2). Also strikingly, opposition to contraception is negatively associated with coresidential relationships. For example, women with high opposition are 15 times less likely to be in Cluster 6 than Cluster 1 (2% versus 30%) whereas women with low opposition are about a third as likely (10% versus 27%). Finally, pregnancy desire is associated with less effective contraceptive use and coresidential relationships. Women who express any desire for pregnancy (versus no desire) have higher odds of membership in Clusters 5 and 6 than Cluster 3 (coresidential relationships and effective contraception).

## 6. Sensitivity results

In our main analyses above, we restricted the sample to women who were not pregnant at baseline and did not become pregnant before completing 12 journals. If a respondent became pregnant after 12 journals, we kept her in the sample but censored her sequence when she first reported being pregnant. We test the implications of these sample restrictions with two robustness checks that rely on alternate sample and sequence state specifications (see Appendix Table E). First, instead of dropping respondents who reported a pregnancy in the first 12 journals and censoring pregnancy states thereafter, we treat pregnancy as a sequence state itself (Appendix Figure E1). This increases our sample to 631 respondents and 25,055 person-weeks. When treating pregnancy as its own state, we arrive at the same six clusters as our original results with only small changes in cluster size and composition, thus suggesting little sensitivity to how we treat pregnancy in the first year of observation (including at baseline). Second, we include the same 631 women in our analysis but ignore pregnancy status over the year of observation (Appendix Figure E2). Again, we find the same joint relationship, sex, and contraceptive trajectories, with slight differences in the size and composition of clusters.

We also test the sensitivity of our results to distinguishing between weeks when respondents used no method of contraception from weeks when they used a less effective method (Appendix F). This creates two additional sequence states: unprotected sex in a non-coresidential relationship (2.3%) and unprotected sex in a coresidential relationship (1.8%). These include relatively small shares of women, and otherwise we again find six joint relationship, sex, and contraceptive trajectories similar to those in our main analysis, with some differences in the size and composition of clusters.

Finally, in results not shown (but available on request), we further examine the sensitivity of our results to including respondents who had more than a three-month gap between journals, standardizing sequence lengths using the longest sequence in the sample, and extending the time frame of our study to include the total number of person-weeks in the panel. The overall conclusions based on these analyses are also consistent with those of our preferred models.

## 7. Discussion

This study offers the first description (to our knowledge) of how young women’s relationship status, sexual activity, and contraceptive use evolve in tandem during the transition to adulthood. Drawing on unique US data and sequence analysis to characterize joint trajectories over a one-year period and cluster analysis to group similar trajectories, we identified six clusters: (1) primarily single; (2) primarily partnered but not coresiding or having sex; (3) transitioning frequently in and out of non-coresidential, sexual relationships while using contraception effectively; (4) transitioning frequently in and out of sexual activity and non-coresidential relationships; (5) primarily having sex using less effective contraception in either non-coresidential or coresidential relationships; and (6) primarily using effective contraception in coresidential relationships. These clusters highlight the diversity of women’s experiences in young adulthood.

One key contribution of this study is to shift away from a single outcome and instead use sequence analysis to provide a contextualized description of the multidimensional processes of relationship, sex, and contraceptive dynamics that unfold over time. This strategy highlighted a substantial amount of instability in the relationship, sex, and contraceptive trajectories characteristic of young adulthood. But it also drew attention to stable trajectories that have received little attention in the literature. Namely, a large minority of women ‒ 43% ‒ were grouped into Clusters 1 and 2 with no or low sexual activity and relatively infrequent transitions between states. Another 9% were in Cluster 6, characterized by relative stability in residential relationships and effective contraception.

Recent research on young adults’ relationship, sexual, and contraceptive behaviors suggests three particularly important sociodemographic patterns related to family formation during the transition to adulthood: (1) Women with lower socioeconomic status are more likely to cohabit at an early age than their more advantaged peers (Sassler, Michelmore, and Holland 2016); (2) casual sex and “hooking up” abounds among college students (though few comparisons with non-college students exist) (England, Shafer, and Fogarty 2008; Manning, Giordano, and Longmore 2006; Stinson 2010); and (3) women with lower socioeconomic status tend to use contraception less consistently and less effectively than women with higher socioeconomic status (England 2016; England et al. 2016). Findings from multinomial models estimating the association between sociodemographic characteristics and cluster membership largely supported these patterns. However, we also found patterns that do not come through clearly in prior research, little of which follows residential, nonresidential, sexual, and nonsexual relationships over time. Notably, we found that it was quite common at this stage of life, irrespective of level of disadvantage, to be in no relationship over the course of a year. This accounted for 27% of women overall and an estimated 32% of those with low levels of disadvantage and 25% with high levels of childhood disadvantage (Cluster 1, Table 4, Model 1).

Consistent with prior research, we found that compared to women with low childhood disadvantage, those with high disadvantage were more likely to be in the cluster characterized by coresiding with a partner and using less effective contraception. Women with low disadvantage were more likely than those with high disadvantage to transition frequently in and out of sex and non-coresidential relationships. The implications of less effective contraceptive use, and any resulting pregnancy, differ substantially in these two relationship contexts. In stable coresidential relationships, contraceptive use may be low because women feel intimately connected to their partners or desire pregnancy (Kusunoki and Upchurch 2011; Wildsmith, Manlove, and Steward-Streng 2015). In less stable non-coresidential relationships, it may be low for reasons unrelated to pregnancy desire, for instance, if sexual relationships are more spontaneous (e.g., one-night stands), if partners communicate poorly or have misgivings about contraception, and/or if women are embedded in a context in which sex without condoms (or other forms of contraception) is normative (Bearak 2014).

Socioeconomic differences in women’s joint trajectories are mediated by their sexual history and outlook toward contraception and fertility. Racial differences, however, remain consistent net of these factors. Relative to non-black women, black women were more likely to belong to a cluster characterized by frequent transitions in relationship status and sex in non-coresidential relationships than one in which they were primarily coresiding with a partner. Extant research indicates lower marriage rates among black women than among white women (Lichter et al. 1992; Raley 1996) and that pregnancies among black women occur less frequently in married or cohabiting unions than pregnancies among white women (Lichter 2012; Raley 2001). Our findings indicate a similar pattern in terms of coresidence and sexual activity: Black women were less likely to be in coresidential relationships than to be single or in nonsexual, non-coresidential relationships compared to non-black women. A quarter of black women were estimated to fall in a cluster characterized by transitions in and out of relationships with a mix of contraceptive efficacy. Such transitions may make it harder to establish consistent contraception, particularly long-lasting contraception, and thus may be associated with higher pregnancy risk. Data constraints in prior studies have often limited demographers’ ability to understand non-coresidential relationships that do not involve pregnancy or childbirth. Our findings illuminate broader differences in the behavioral trajectories that give rise to racial differences in the relationship contexts surrounding conception.

Sexual history, in terms of respondents’ number of sexual partners before baseline but not in terms of age of sexual debut, was also highly predictive of which cluster women belonged to. That is, women who had greater numbers of previous partners had higher odds of following a joint trajectory that included sexual relationships. This finding is consistent with the life course perspective, which posits that earlier life events, including earlier sexual experiences, set the stage for subsequent sexual and relationship trajectories. However, among those who followed a trajectory dominated by time spent in relationships, number of previous partners was not predictive of whether their trajectory also included effective contraception or coresidence. This may reflect unobserved heterogeneity in the extent to which women’s earlier sexual experiences were accompanied by contraception and/or coresidence ‒ a possibility we were unable to explore given that information on these experiences was not collected in the RDSL.

Contraceptive attitudes were also an important predictor of which cluster women belonged to. Women who possessed more negative attitudes toward contraception were more likely to be in a cluster characterized by less effective contraception and less likely to be in one characterized by protected sex in coresidential relationships. This finding is consistent with previous research showing a strong relationship between contraceptive attitudes and contraceptive use among sexually active women (Frost, Singh, and Finer 2007). Desire for pregnancy was another strong predictor of the path women followed. In particular, pro-pregnancy desire was associated with higher odds of being in a coresidential relationship and/or using less effective contraception relative to using effective contraception in a non-coresidential relationship.

The RDSL data provide fine-grained, prospective information on a critical life stage characterized by frequent transitions. Despite the unique insights gained by exploring the co-evolution of relationships, sexual activity, and contraceptive use, this study faces limitations. Our approach limits to some extent the complexity we can make sense of in our categories of relationships and contraception. We thus potentially collapse over meaningful differences, for example, not distinguishing between consistent condom use and more effective hormonal methods in our measure of contraceptive use. This may overstate effective contraceptive use, particularly among groups with high rates of condom use.

The targeted RDSL sample is another limitation. While the RDSL sample is representative of 18 to 19 year old women in one Michigan county, the racial demographics of the county diverge from those of the United States overall. The extent to which our findings are generalizable to other racial and ethnic groups in the United States (e.g., Hispanics), to young men, or to other contexts remains an open question.

We propose an approach for examining how relationships unfold during this period of relative instability in the life course. How young women’s relationship, sexual, and contraceptive trajectories co-evolve have important implications for early family formation and the contexts in which this formation takes place. Examining the joint progression of these behaviors together not only reflects their intertwined nature but also further reveals the diversity of paths that characterize young women’s lived experiences and facilitates a more holistic understanding of the processes by which demographic differences in early family formation take shape.

## Figures and Tables

**Table 1:** Relationship, sex, and contraception categories for sequence analysis, RDSL, N = 581

Relationship	Sex and contraception	Sequence state	Person-weeks in this state (N = 22,389)
Not in a relationship	No sex	0	39.20%
In a relationship, not coresiding	No sex	1	25.95%
In a relationship, not coresiding	Sex and no protection, inconsistent condom use, diaphragm, spermicide, withdrawal, or rhythm	2	6.86%
In a relationship, not coresiding	Sex and consistent condom use, pill, patch, ring, shot, implant, or IUD	3	15.12%
In a relationship, coresiding	Sex and no protection, inconsistent condom use, diaphragm, spermicide, withdrawal, or rhythm	4	4.30%
In a relationship, coresiding	Sex and consistent condom use, pill, patch, ring, shot, implant, or IUD	5	8.58%

**Table 2:** Descriptive statistics of analytic sample, RDSL, N = 581

	Mean
*Sociodemographic background*	
Childhood disadvantage	
Low	.40
Medium	.28
High	.32
Race	
Non-black	.73
Black	.27
*Employment and education*	
Employment status	
Unemployed	.50
Employed	.50
Educational status	
Incomplete H.S.	.17
Graduated H.S., not enrolled in school	.17
Enrolled in post-secondary school	.66
*Sexual and reproductive history*	
Age at sexual debut	
Sexual debut >14 years	.88
Early sexual debut (≤14)	.12
# sex partners by baseline	
0	.33
1	.18
2‒3	.25
4 or more	.24
Pregnancy history	
No pregnancy by baseline	.86
Had pregnancy by baseline	.14
*Attitudes toward sex, contraception, and fertility*	
Nonmarital sex	
Opposed	.51
Not opposed	.49
Contraceptive opposition	
Low	.29
Mild	.35
Moderate	.17
High	.19
Belief: babies improve relationships	
Disagree	.82
Agree	.18
Pregnancy desire	
Does not desire pregnancy	.94
Desires pregnancy	.06

*Notes*: Sample includes women in the study for at least one year, without a gap between journals of more than three months, who were not pregnant at baseline and did not become pregnant before completing 12 journals.

**Table 3:** Description of sequence clusters, RDSL, N = 581

	Cluster 1	Cluster 2	Cluster 3	Cluster 4	Cluster 5	Cluster 6
	No relationship	Non-coresidential, no sex	Non-coresidential, effective	Mixed relationship and sex	Less effective	Coresidential, effective
% of pers% of person-weeks in cluster with:						
No relationship	89	13	6	44	7	2
Not coresiding, no sex	8	76	26	28	14	10
Not coresiding, less effective	1	3	7	6	31	2
Not coresiding, effective	2	8	59	17	8	10
Coresiding, less effective	<1	<1	1	1	28	6
Coresiding, effective	<1	1	2	4	12	71
Mean elements in cluster	2.07	2.70	3.33	3.52	3.94	2.92
Mean episodes in cluster	4.82	8.51	14.77	13.41	13.31	7.01
Mean gap between journals in cluster	1.17	1.25	1.25	1.41	1.36	1.32
Average sequence length	44.87	42.68	42.71	38.50	38.59	40.62
Person-weeks	6,865	3,628	3,305	3,966	2,750	1,875
Share of person-weeks	30.7%	16.2%	14.8%	17.7%	12.3%	8.4%
Women	158	92	82	117	81	51
Share of women	27.2%	15.8%	14.1%	20.1%	13.9%	8.8%

**Table 4:** Predicted probabilities from multinomial logistic regressions examining correlates of sequence cluster membership, RDSL, N = 581

	Cluster 1	Cluster 2	Cluster 3	Cluster 4	Cluster 5	Cluster 6
	No relationship (27.2%)	Non-coresidential, no sex (15.8%)	Non-coresidential, effective (14.1%)	Mixed relationship and sex (20.1%)	Less effective (13.9%)	Coresidential, effective (8.8%)
**Model 1**												
*Sociodemographic background*												
Childhood disadvantage												
Low	.32	.19	.17	.18	.08	.07
Medium	.23	.13	.14	.24	.17	.08
High	.25	.15	.10	.19	.20	.11
Race												
Non-black	.27	.16	.15	.17	.16	.10
Black	.28	.16	.12	.28	.10	.05
**Model 2**												
*Sociodemographic background*												
Childhood disadvantage												
Low	.28	.17	.15	.19	.12	.09
Medium	.24	.13	.15	.25	.16	.08
High	.30	.17	.12	.18	.14	.10
Race												
Non-black	.26	.16	.15	.17	.17	.10
Black	.30	.16	.12	.29	.09	.05
*Employment and education*												
Employment status												
Unemployed	.28	.17	.14	.18	.15	.08
Employed	.26	.15	.14	.23	.13	.10
Educational status												
Incomplete H.S.	.25	.11	.10	.30	.16	.09
Graduated H.S., not enrolled	.30	.13	.12	.17	.17	.12
Enrolled post-secondary	.27	.18	.16	.19	.12	.08
*Sexual and reproductive history*												
Age at sexual debut												
Sexual debut (>14 years)	.27	.16	.15	.21	.13	.08
Early sexual debut (≤14)	.28	.13	.11	.18	.17	.12
# sex partners by baseline												
0	.56	.21	.04	.16	.01	.02
1	.14	.20	.23	.16	.13	.15
2–3	.19	.12	.21	.22	.17	.10
4 or more	.09	.10	.17	.30	.24	.10
Pregnancy history												
No pregnancy by baseline	.27	.15	.15	.21	.14	.08
Had pregnancy by baseline	.26	.19	.10	.17	.15	.12
*Attitudes toward sex, contraception, and fertility*												
Nonmarital sex												
Opposed	.28	.17	.12	.22	.13	.08
Not opposed	.27	.14	.16	.19	.15	.09
Contraceptive opposition												
Low	.27	.16	.19	.19	.09	.10
Mild	.26	.18	.11	.22	.12	.12
Moderate	.27	.14	.13	.16	.20	.08
High	.30	.13	.13	.22	.19	.02
Belief: babies improve rels.												
Disagree	.27	.17	.13	.20	.14	.09
Agree	.29	.12	.18	.21	.12	.09
Pregnancy desire												
Does not desire pregnancy	.27	.16	.15	.21	.13	.08
Desires pregnancy	.25	.16	.04	.14	.25	.16

*Notes*: All models include controls for religiosity. Full models shown in Appendix Table D.

**Table 5:** Summary results of multinomial logistic regressions examining correlates of sequence cluster membership, RDSL, N = 581

	Cluster 1	Cluster 2	Cluster 3	Cluster 4	Cluster 5	Cluster 6
	No relationship	Non-coresidential, no sex	Non-coresidential, effective	Mixed relationship and sex	Less effective	Coresidential, effective
**Model 1**						
*Sociodemographic background*						
Childhood disadvantage (v. low)						
Medium	+4,5	+4,5	+5	−1,2	−1,2,3	
High	+5	+5	+5,6	+5	−1,2,3,4	−3
Black				−5,6	+4	+4
**Model 2**						
*Sociodemographic background*						
Childhood disadvantage (v. low)						
Medium						
High						
Black	−5			−5.6	+1,4	+4
*Employment and education*						
Employed						
Educational status (v. incomplete H.S.)						
Graduated H.S., not enrolled						
Enrolled in postsecondary		−4	−4	+2,3		
*Sexual and reproductive history*						
Early sexual debut (≤14)						
# sex partners by baseline (v. 0)						
1	+2,3,4,5,6	−1 & +3,5,6	−1,2,4	−1 & +3,5,6	−1,2,4	−1,2,4
2–3	+3,4,5,6	+3,4,5,6	−1,2,4	−1,2 & +3,5	−1,2,4	−1,2
4 or more	+3,4,5,6	+3,4,5,6	−1,2	−1,2 & +5	−1,2,4	−1,2
Had pregnancy by baseline						
*Attitudes toward sex, contraception, and fertility*						
Not opposed to nonmarital sex						
Contraceptive opposition (v. low)						
Mild			+5		−3	
Moderate	+5	+5	+5	+5	−1,2,3,4	
High	−6		+5	−6	−3,6	+1,4,5
Belief: babies improve rels.						
Desires pregnancy			+5,6	+5	−3,4	−3

*Notes*: All models include controls for religiosity. Full models shown in Appendix Table D. Numbers indicate statistically significant contrasts (p<.05). +/− indicates that the direction of difference.

**Figure C:**
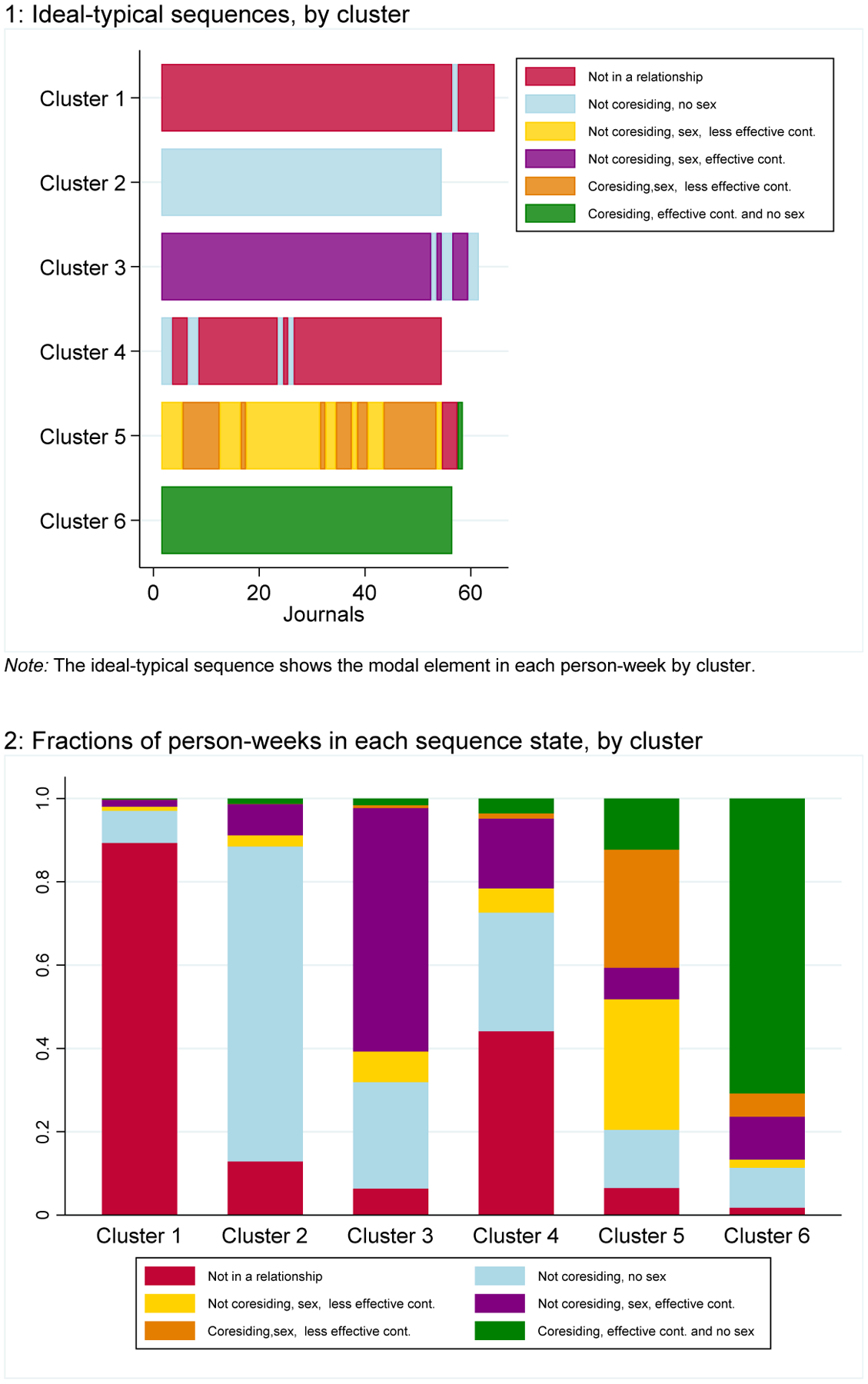
Supplemental figures describing six clusters of relationship-sex-contraception sequences in the first year of the RDSL, N = 581

**Figure E:**
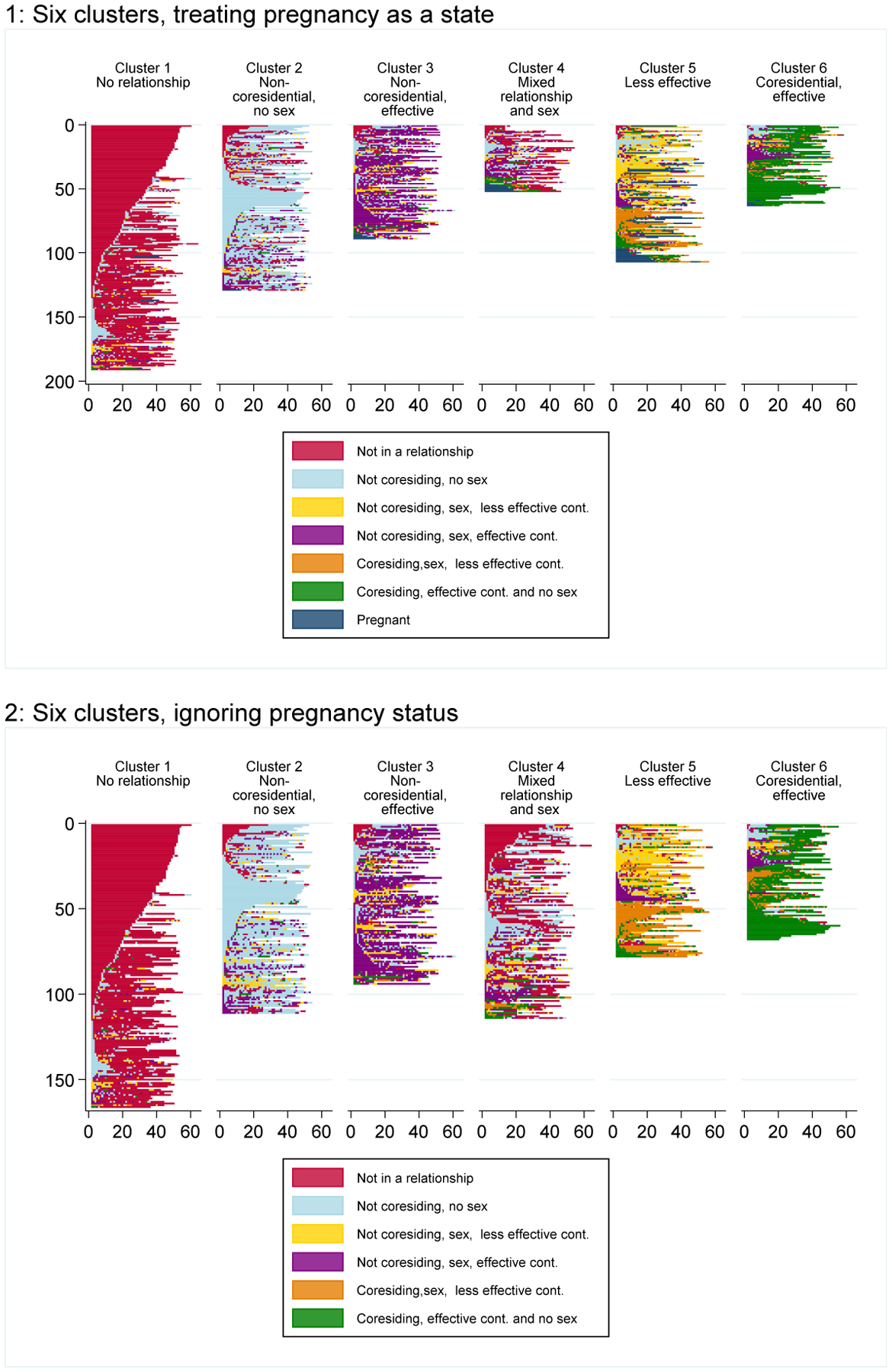
Robustness checks using alternate sample and sequence state specifications, RDSL, N = 631

**Figure F:**
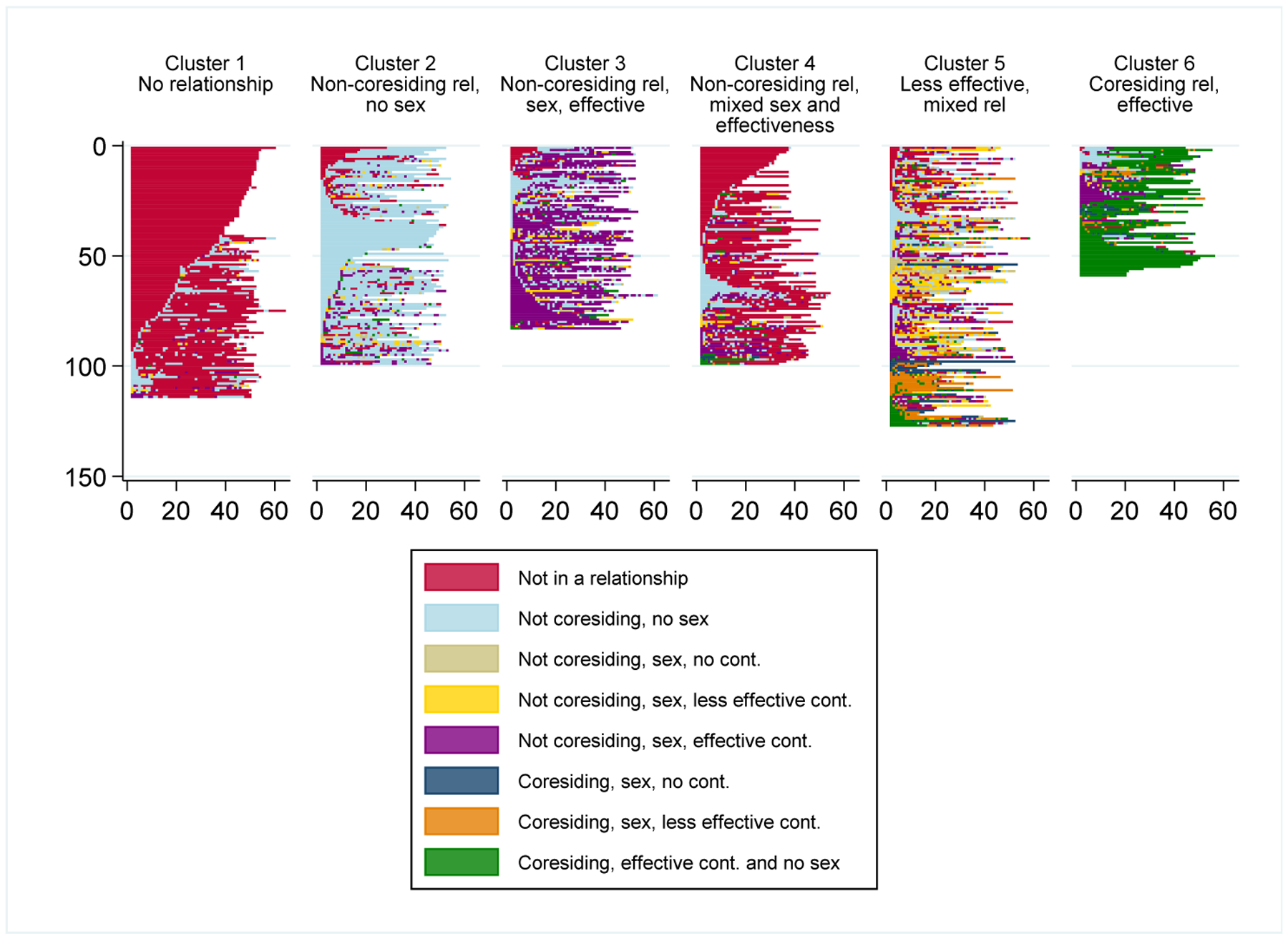
Robustness check distinguishing no contraceptive method from less effective methods, RDSL, N = 581

## Footnotes

5 These short intervals between journals minimized recall bias, while online and automated phone participation were intended to reduce social desirability bias (Catania et al. 1990; Turner et al. 1998; Barber, Kusunoki, and Gatny 2011).

6 When respondents had a gap of 14 or more days since their last journal, sex and relationship questions were asked about the prior seven days.

7 We refer to coresiding rather than cohabiting because we combine married, engaged, and cohabiting respondents. This decision reflects that respondents were only married or engaged in 8.3% of journals in our analytic sample.

8 A total of 97% of respondents identify as either black or white. There were therefore too few respondents of other races to create additional, meaningful categories. We use black instead of African American because this group includes Hispanic blacks (2.6% of the final sample). The non-black group is 90% white, 7% Hispanic, 1% Asian/API, and 2% Native American.

9 Respondents completed 12 to 64 journals in the first year of the study. We drop the first week because information about sex and contraception from the prior week was not collected during the baseline interview, so sequence lengths range from 11 to 63.

## Appendix

**Table A:** Patterns of study participation among RDSL respondents in the first year of the study

	All RDSL	Analytic sample
Mean number of journals	28.98	39.54
% <12	24	0
% 12 to 24	18	14
% 25 to 49	44	65
% 50 and above	13	22
Mean gap in weeks	1.79	1.40
% on time	75	89
%> 2 to 4 weeks	18	11
% >4 to 8 weeks	6	1
% > 8 to 12 weeks	1	0
% over 12 weeks	1	0
Mean of maximum gap	6.30	4.01
% on time	36	44
%> 2 to 4 weeks	23	26
% >4 to 8 weeks	9	11
% > 8 to 12 weeks	13	14
% over 12 weeks	18	5
Women	992	581
Person-weeks	28,755	22,389

**Table B:** Descriptive statistics of RDSL

	All respondents (*N*=992)	Analytic sample (*N*=581)
*Sociodemographic background*		
Childhood disadvantage		
Low	.31	.40
Medium	.27	.28
High	.41	.32
Race		
Non-black	.67	.73
Black	.34	.27
*Employment and education*		
Employment status		
Unemployed	.50	.50
Employed	.50	.50
Educational status		
Incomplete H.S.	.22	.17
Graduated H.S., not enrolled in school	.22	.17
Enrolled in post-secondary	.56	.66
*Sexual and reproductive history*		
Age at sexual debut		
Sexual debut >14 years	.83	.88
Early sexual debut (≤14)	.17	.12
# sex partners by baseline		
0	.23	.33
1	.17	.18
2–3	.27	.25
4 or more	.33	.24
Pregnancy history		
No pregnancy by baseline	.74	.86
Had pregnancy by baseline	.26	.14
*Attitudes toward sex, contraception, and fertility*		
Nonmarital sex		
Opposed	.52	.51
Not opposed	.48	.49
Contraceptive opposition		
Low	.24	.29
Mild	.34	.35
Moderate	.19	.17
High	.22	.19
Belief: babies improve relationships		
Disagree	.82	.82
Agree	.18	.18
Pregnancy desire		
Does not desire pregnancy	.90	.94
Desires pregnancy	.10	.06

**Table D:** Results of adjusted multinomial logistic regressions examining correlates of sequence cluster membership and sociodemographic background, relative risk ratios, RDSL, N = 581

	Model 1						Model 2					
	Cluster 1	Cluster 2	Cluster 3	Cluster 4	Cluster 5	Cluster 6	Cluster 1	Cluster 2	Cluster 3	Cluster 4	Cluster 5	Cluster 6
	No relationship	Non-coresidential, no sex	Non-coresidential, effective	Mixed relationship and sex	Less effective	Coresidential, effective	No relationship	Non-coresidential, no sex	Non-coresidential, effective	Mixed relationship and sex	Less effective	Coresidential, effective
**CLUSTER 1 REFERENCE**												
*Sociodemographic background*												
Childhood disadvantage (v. low)												
Medium	--	0.91	1.16	1.88*	3.12**	1.57	--	0.88	1.20	1.63	1.68	1.05
High	--	0.97	0.76	1.37	3.39***	1.94	--	0.89	0.67	0.89	1.07	1.00
Black	--	1.00	0.81	1.58	0.62	0.48	--	0.82	0.59	1.33	0.36*	0.38
*Employment and education*												
Employed								0.92	1.17	1.41	0.93	1.36
Educational status (v. incomplete H.S.)												
Graduated H.S., not enrolled							--	0.96	0.98	0.43	0.82	1.11
Enrolled in postsecondary							--	1.39	1.41	0.52	0.58	0.76
*Sexual and reproductive history*												
Early sexual debut (≤14)							--	0.83	0.80	0.90	1.40	1.61
# sex partners by baseline (v. 0)												
1							--	4.04**	29.32***	4.11**	42.99***	31.54***
2–3							--	1.80	18.82***	4.18***	40.27***	15.08***
4 or more							--	2.72	29.62***	11.48***	123.45***	31.86***
Had pregnancy by baseline							--	1.31	0.69	0.86	1.14	1.55
*Attitudes toward sex, contraception, and fertility*												
Not opposed to nonmarital sex							--	0.88	1.61	0.96	1.27	1.24
Contraceptive opp. (v. low)												
Mild							--	1.22	0.58	1.20	1.51	1.25
Moderate							--	0.90	0.75	0.89	2.71*	0.93
High							--	0.72	0.59	1.04	2.00	0.17*
Belief: babies improve rels.							--	0.64	1.29	0.98	0.73	0.93
Desires pregnancy							--	1.18	0.29	0.83	2.93	2.80
Constant	--	0.46**	0.64	0.53*	0.32***	0.38**	--	0.26*	0.04***	0.30*	0.01***	0.04***
**CLUSTER 2 REFERENCE**												
*Sociodemographic background*												
Childhood disadvantage (v. low)												
Medium	1.10	--	1.27	2.06*	3.43**	1.73	1.14	--	1.37	1.85	1.91	1.20
High	1.03	--	0.78	1.41	3.49**	1.99	1.12	--	0.75	0.99	1.19	1.12
Black	1.00	--	0.81	1.58	0.62	0.48	1.22	--	0.72	1.62	0.44	0.47
*Employment and education*												
Employed							1.09		1.28	1.54	1.02	1.49
Educational status (v. incomplete H.S.)												
Graduated H.S., not enrolled							1.05	--	1.03	0.45	0.86	1.17
Enrolled in postsecondary							0.72	--	1.02	0.38*	0.42	0.55
*Sexual and reproductive history*												
Early sexual debut (≤14)							1.21	--	0.96	1.09	1.69	1.94
# sex partners by baseline (v. 0)												
1							0.25**	--	7.26***	1.02	10.65**	7.81**
2–3							0.56	--	10.48***	2.33*	22.43***	8.40**
4 or more							0.37	--	10.90***	4.23**	45.43***	11.72**
Had pregnancy by baseline							0.76	--	0.53	0.66	0.87	1.19
*Attitudes toward sex, contraception, and fertility*												
Not opposed to nonmarital sex							1.13	--	1.83	1.09	1.43	1.40
Contraceptive opp. (v. low)												
Mild							0.82	--	0.48	0.99	1.24	1.03
Moderate							1.11	--	0.83	0.99	3.01*	1.03
High							1.39	--	0.82	1.44	2.78	0.24
Belief: babies improve rels.							1.56	--	2.01	1.52	1.14	1.44
Desires pregnancy							0.85	--	0.25	0.70	2.48	2.37
Constant	2.19**	--	1.40	1.16	0.70	0.84	3.79*	--	0.16*	1.15	0.06**	0.13*
**CLUSTER 3 REFERENCE**												
*Sociodemographic background*												
Childhood disadvantage												
(v. low)												
Medium	0.86	0.79	--	1.62	2.70*	1.36	0.83	0.73	--	1.35	1.39	0.88
High	1.31	1.28	--	1.80	4.46***	2.54*	1.49	1.33	--	1.32	1.59	1.50
Black	1.24	1.24	--	1.96	0.77	0.59	1.71	1.40	--	2.27	0.61	0.65
*Employment and education*												
Employed							0.86	0.78		1.21	0.80	1.16
Educational status (v. incomplete H.S.)												
Graduated H.S., not enrolled							1.02	0.98	--	0.44	0.84	1.14
Enrolled in postsecondary							0.71	0.98	--	0.37*	0.41	0.54
*Sexual and reproductive history*												
Early sexual debut (≤14)							1.26	1.04	--	1.14	1.76	2.02
# sex partners by baseline (v. 0)												
1							0.03***	0.14***	--	0.14***	1.47	1.08
2–3							0.05***	0.10***	--	0.22**	2.14	0.80
4 or more							0.03***	0.09***	--	0.39	4.17	1.08
Had pregnancy by baseline							1.45	1.90	--	1.25	1.65	2.25
*Attitudes toward sex, contraception, and fertility*												
Not opposed to nonmarital sex							0.62	0.55	--	0.60	0.79	0.77
Contraceptive opp. (v. low)												
Mild							1.73	2.10	--	2.07	2.61*	2.16
Moderate							1.34	1.21	--	1.20	3.63*	1.25
High							1.68	1.21	--	1.75	3.38*	0.29
Belief: babies improve rels.							0.77	0.50	--	0.75	0.57	0.72
Desires pregnancy							3.40	4.01	--	2.81	9.95*	9.52*
Constant	1.56	0.71	--	0.83	0.50*	0.60	23.38***	6.16*	--	7.10**	0.34	0.82
**CLUSTER 4 REFERENCE**												
*Sociodemographic background*												
Childhood disadvantage (v. low)												
Medium	0.53*	0.49*	0.62	--	1.66	0.84	0.61	0.54	0.74	--	1.03	0.65
High	0.73	0.71	0.55	--	2.47*	1.41	1.13	1.01	0.75	--	1.20	1.13
Black	0.63	0.63	0.51	--	0.40*	0.30*	0.75	0.62	0.44	--	0.27**	0.29*
*Employment and education*												
Employed							0.71	0.65	0.83		0.66	0.96
Educational status												
(v. incomplete H.S.)												
Graduated H.S., not enrolled							2.34	2.23	2.29	--	1.92	2.60
Enrolled in postsecondary							1.91	2.65*	2.71*	--	1.10	1.46
*Sexual and reproductive history*												
Early sexual debut (≤14)							1.11	0.92	0.88	--	1.55	1.78
# sex partners by baseline (v. 0)												
1							0.24**	0.98	7.13***	--	10.46**	7.67**
2–3							0.24***	0.43*	4.50**	--	9.63**	3.61
4 or more							0.09***	0.24**	2.58	--	10.75**	2.77
Had pregnancy by baseline							1.16	1.52	0.80	--	1.32	1.80
*Attitudes toward sex, contraception, and fertility*												
Not opposed to nonmarital							1.04	0.92	1.68	--	1.32	1.29
sex												
Contraceptive opp. (v. low)												
Mild							0.83	1.01	0.48	--	1.26	1.04
Moderate							1.12	1.01	0.83	--	3.03*	1.04
High							0.96	0.69	0.57	--	1.93	0.16*
Belief: babies improve rels.							1.03	0.66	1.33	--	0.75	0.95
Desires pregnancy							1.21	1.43	0.36	--	3.54*	3.39
Constant	1.89*	0.86	1.21	--	0.61	0.73	3.29*	0.87	0.14**	--	0.05**	0.12*
**CLUSTER 5 REFERENCE**												
*Sociodemographic background*												
Childhood disadvantage (v. low)												
Medium	0.32**	0.29**	0.37*	0.60	--	0.50	0.60	0.52	0.72	0.97	--	0.63
High	0.29***	0.29**	0.22***	0.40*	--	0.57	0.94	0.84	0.63	0.83	--	0.94
Black	1.60	1.60	1.29	2.53*	--	0.77	2.80*	2.29	1.64	3.71**	--	1.07
*Employment and education*												
Employed							1.07	0.98	1.25	1.52		1.46
Educational status												
(v. incomplete H.S.)												
Graduated H.S., not enrolled							1.22	1.16	1.19	0.52	--	1.36
Enrolled in postsecondary							1.73	2.40	2.45	0.91	--	1.32
*Sexual and reproductive history*												
Early sexual debut (≤14)							0.72	0.59	0.57	0.65	--	1.15
# sex partners by baseline v. 0)												
1							0.02***	0.09**	0.68	0.10**	--	0.73
2–3							0.02***	0.04***	0.47	0.10**	--	0.37
4 or more							0.01***	0.02***	0.24	0.09**	--	0.26
Had pregnancy by baseline							0.88	1.15	0.61	0.76	--	1.36
*Attitudes toward sex, contraception, and fertility*												
Not opposed to nonmarital							0.79	0.70	1.27	0.76	--	0.98
sex												
Contraceptive opp. (v. low)												
Mild							0.66	0.80	0.38*	0.79	--	0.82
Moderate							0.37*	0.33*	0.28*	0.33*	--	0.34
High							0.50	0.36	0.30*	0.52	--	0.08**
Belief: babies improve rels.							1.37	0.88	1.77	1.33	--	1.27
Desires pregnancy							0.34	0.40	0.10*	0.28*	--	0.96
Constant	3.10***	1.42	1.99*	1.65	--	1.20	68.11***	17.95**	2.91	20.68**	--	2.39
**CLUSTER 6 REFERENCE**												
*Sociodemographic background*												
Childhood disadvantage (v. low)												
Medium	0.64	0.58	0.73	1.19	1.98	--	0.95	0.83	1.14	1.54	1.59	--
High	0.52	0.50	0.39*	0.71	1.75	--	1.00	0.89	0.67	0.88	1.06	--
Black	2.09	2.09	1.69	3.30*	1.30	--	2.62	2.14	1.53	3.48*	0.94	--
*Employment and education*												
Employed							0.73	0.67	0.86	1.04	0.68	
Educational status												
(v. incomplete H.S.)												
Graduated H.S., not enrolled							0.90	0.86	0.88	0.38	0.74	--
Enrolled in postsecondary							1.31	1.82	1.86	0.69	0.76	--
*Sexual and reproductive history*												
Early sexual debut (≤14)							0.62	0.52	0.50	0.56	0.87	--
# sex partners by baseline (v. 0)												
1							0.03***	0.13**	0.93	0.13**	1.36	--
2–3							0.07***	0.12**	1.25	0.28	2.67	--
4 or more							0.03***	0.09**	0.93	0.36	3.87	--
Had pregnancy by baseline							0.64	0.84	0.44	0.56	0.73	--
*Attitudes toward sex, contraception, and fertility*												
Not opposed to nonmarital							0.81	0.71	1.30	0.78	1.02	--
sex												
Contraceptive opp. (v. low)												
Mild							0.80	0.98	0.46	0.96	1.21	--
Moderate							1.07	0.97	0.80	0.96	2.91	--
High							5.88*	4.24	3.49	6.11*	11.78**	--
Belief: babies improve rels.							1.08	0.69	1.39	1.05	0.79	--
Desires pregnancy							0.36	0.42	0.10*	0.29	1.04	--
Constant	2.60**	1.19	1.67	1.38	0.84	--	28.49***	7.51*	1.22	8.65*	0.42	--

*Notes*: All models include controls for religiosity.

**Table E:** Robustness checks using alternate sample and sequence state specifications, RDSL, N = 631

	E1: Six clusters, treating pregnancy as a state						E2. Six clusters, ignoring pregnancy status					
	Cluster 1	Cluster 2	Cluster 3	Cluster 4	Cluster 5	Cluster 6	Cluster 1	Cluster 2	Cluster 3	Cluster 4	Cluster 5	Cluster 6
% of person-weeks in cluster with:												
No relationship	71	19	11	19	7	3	69	16	11	35	6	4
Not coresiding, no sex	18	67	24	27	17	14	21	63	30	34	13	10
Not coresiding, ineffective	5	3	7	8	19	6	4	6	9	11	17	7
Not coresiding, effective	4	10	47	25	17	24	4	14	46	11	22	19
Coresiding, ineffective	<1	<1	1	<1	19	5	<1	<1	1	4	28	16
Coresiding, effective	2	<1	6	8	11	41	<1	<1	4	5	14	43
Pregnancy	<1	<1	4	13	11	6						
*Sociodemographic background*												
Childhood disadvantage												
Low	.44	.49	.42	.27	.21	.35	.42	.44	.44	.39	.23	.31
Medium	.24	.23	.31	.37	.32	.32	.26	.22	.30	.32	.31	.31
High	.32	.28	.27	.37	.48	.33	.33	.34	.27	.29	.46	.38
Race												
Non-black	.72	.70	.78	.58	.76	.84	.72	.68	.80	.61	.79	.87
Black	.28	.30	.22	.42	.24	.16	.28	.32	.20	.39	.21	.13
Share of women	30.3%	20.4%	14.1%	8.2%	17.0%	10.0%	26.3%	17.6%	14.9%	18.1%	12.4%	10.8%

**Table F:** Robustness check distinguishing no contraceptive method from less effective methods, RDSL, N = 581

	Cluster 1	Cluster 2	Cluster 3	Cluster 4	Cluster 5	Cluster 6
% of person-weeks in cluster with: No relationship	89	13	6	70	20	3
Not coresiding, no sex	10	75	25	16	20	9
Not coresiding, no cont.	<1	1	2	<1	8	1
Not coresiding, less effective	<1	12	5	2	15	1
Not coresiding, less effective	1	7	59	9	13	10
Coresiding, no cont	0	<1	<1	<1	7	4
Coresiding, less effective cont	0	<1	<1	<1	11	4
Coresiding, effective cont	<1	1	2	2	6	68
*Sociodemographic background*						
Childhood disadvantage						
Low	0.50	0.48	0.54	0.42	0.30	0.37
Medium	0.24	0.23	0.28	0.26	0.35	0.28
High	0.25	0.30	0.19	0.32	0.35	0.35
Race						
Non-black	0.80	0.71	0.83	0.65	0.69	0.88
Black	0.20	0.29	0.17	0.35	0.31	0.12
Share of women	19.6%	17.0%	14.3%	17.0%	21.9%	10.2%
